# Research progress of traditional Chinese medicine in improving hepatic fibrosis based on inhibiting pathological angiogenesis

**DOI:** 10.3389/fphar.2023.1303012

**Published:** 2023-12-12

**Authors:** Zhen Li, Junfeng Zhu, Hao Ouyang

**Affiliations:** Yueyang Hospital of Integrated Traditional Chinese and Western Medicine, Shanghai University of Traditional Chinese Medicine, Shanghai, China

**Keywords:** hepatic fibrosis, pathological angiogenesis, traditional Chinese medicine monomer, single herbal extract, traditional Chinese medicine formula

## Abstract

Hepatic fibrosis is the formation of scar tissue in the liver. This scar tissue replaces healthy liver tissue and can lead to liver dysfunction and failure if left untreated. It is usually caused by chronic liver disease, such as hepatitis B or C, alcohol abuse, or non-alcoholic fatty liver disease. Pathological angiogenesis plays a crucial role in the development of hepatic fibrosis by promoting the growth of new blood vessels in the liver. These new vessels increase blood flow to the damaged areas of the liver, which triggers the activation of hepatic stellate cells (HSCs). HSCs are responsible for producing excess collagen and other extracellular matrix proteins that contribute to the development of fibrosis. Pathological angiogenesis plays a crucial role in the development of hepatic fibrosis by promoting the growth of new blood vessels in the liver. These new vessels increase blood flow to the damaged areas of the liver, which triggers the activation of HSCs. HSCs are responsible for producing excess collagen and other extracellular matrix proteins that contribute to the development of fibrosis. Traditional Chinese medicine (TCM) has been found to target pathological angiogenesis, thereby providing a potential treatment option for hepatic fibrosis. Several studies have demonstrated that TCM exhibits anti-angiogenic effects by inhibiting the production of pro-angiogenic factors, such as vascular endothelial growth factor and angiopoietin-2, and by reducing the proliferation of endothelial cells. Reviewing and highlighting the unique TCM recognition of treating hepatic fibrosis by targeting pathological angiogenesis may shed light on future hepatic fibrosis research.

## 1 Introduction

Hepatic fibrosis is a chronic liver disease characterized by the accumulation of excess collagen in the liver tissues, leading to scarring and ultimately, loss of liver function (Friedman and Pinzani, 2022). This condition is a major cause of morbidity and mortality worldwide, affecting millions of people each year (Schwabe et al., 2020). The prevalence of hepatic fibrosis varies by region and demographic, but it is estimated to be increasing globally due to the rise in risk factors such as alcohol consumption, obesity, viral hepatitis, and metabolic syndrome (Parola and Pinzani, 2019). Approximately 10% of the world’s population is estimated to be affected by hepatic fibrosis (Schwabe et al., 2020). In some countries, such as Egypt, the Middle East, and Africa, the prevalence is even higher due to the high incidence of viral hepatitis and other risk factors (Mahmud et al., 2022). Hepatic fibrosis can occur through various mechanisms, including injury or inflammation to the liver cells, chronic exposure to toxins or drugs, or viral infections such as hepatitis B or C (Gines et al., 2022). Over time, these insults gradually induce hypoxia and oxidative stress, while also stimulating the secretion of growth factors and activating immune cells. This creates an inflammatory response and triggers hypoxia, ultimately leading to the activation of hepatic stellate cells (HSCs). These HSCs are responsible for the production of collagen fibers, which contribute to the formation of scar tissue (Baghaei et al., 2022).

While there are several treatments available for hepatic fibrosis, there are also several limitations to these treatments. One of the limitations of current treatment of hepatic fibrosis is that it often focuses on managing symptoms rather than addressing the underlying cause of the condition (Neshat et al., 2021). Another limitation is that they are not always effective for all patients (Guo and Lu, 2020). For some patients, medications may not work as well as they should, or they may experience unwanted side effects that make them difficult to tolerate. Cost is another limitation of current treatments for hepatic fibrosis. In summary, while there are several treatments available for hepatic fibrosis, there are also several limitations to these treatments. More research is needed to develop new and more effective treatments that address the underlying causes of hepatic fibrosis and are accessible to all patients.

Studies have found that pathological angiogenesis can promote the progression of hepatic fibrosis. The formation of new blood vessels not only provides sufficient oxygen and nutrients, but also provides necessary conditions for the proliferation of fibroblasts and synthesis of collagen fibers (Medina et al., 2004). In addition, new blood vessels can directly stimulate the transformation of liver cells into fibroblasts by releasing growth factors and cytokines, thereby promoting the progression of hepatic fibrosis (Affo and Sancho-Bru, 2014). Therefore, blocking pathological angiogenesis may help to slow down the progression of hepatic fibrosis and provide new strategies for its treatment.

In recent years, traditional Chinese medicine (TCM) has gained attention for its potential to treat various chronic diseases, including hepatic fibrosis. Many TCM monomers and single herbal extracts, and TCM formulas have been shown to have anti-angiogenesis, anti-inflammatory, antioxidant, and immunomodulatory effects that may be beneficial for liver health (Li, 2020). However, there is a lack of systematic organization in this area. Therefore, this article systematically reviews the mechanism by which TCM monomers, single herbal extracts and TCM formulas prevent and treat hepatic fibrosis by targeting and inhibiting the formation of pathological blood vessels. This is the innovation of this article, which helps to reveal the mechanism of action of TCM monomers, single herbal extracts and TCM formulas for the prevention and treatment of hepatic fibrosis. What’s more, it helps to strengthen the scientific basis for TCM’s use in treating hepatic fibrosis and may prompt further research into new drug development and treatment strategies.

## 2 Mechanisms underlying the pathogenesis of hepatic fibrosis

The process of hepatic fibrosis is closely related to the activation of HSCs, which are also known as Ito cells, lipocytes, or perisinusoidal cells (Higashi et al., 2017). HSCs are predominantly located in the space of Disse, which is an area between hepatocytes and sinusoids (Puche et al., 2013). In the quiescent state, HSCs store vitamin A in their cytoplasmic droplets and play an important role in liver homeostasis by regulating the uptake and storage of retinol, synthesizing extracellular matrix (ECM) components such as collagen, and secreting growth factors that influence cell proliferation and differentiation (Kamm and McCommis, 2022).

**FIGURE 1:**
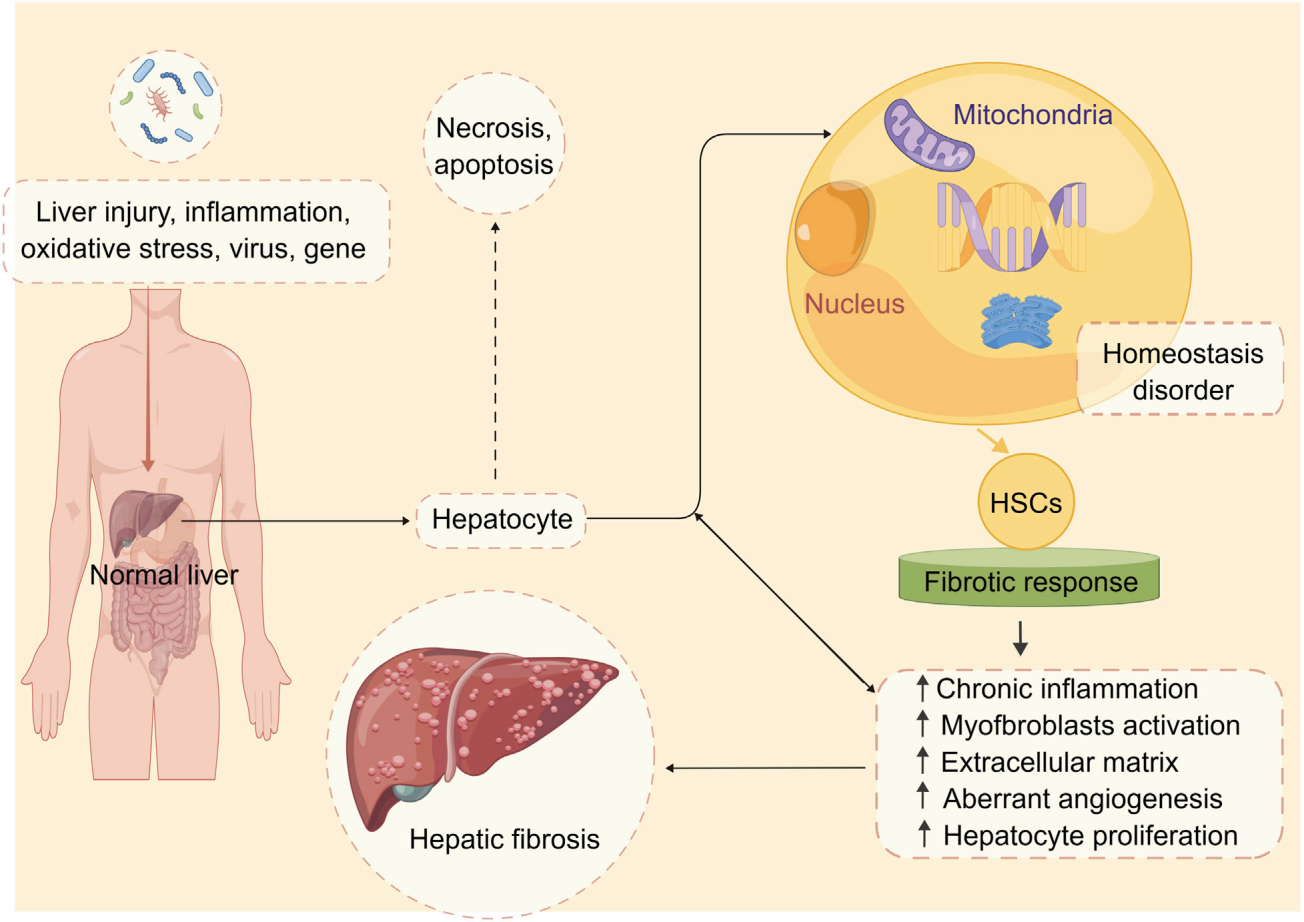
The pathophysiology of hepatic fibrosis. Under the stimulation of multiple factors, the stable state of liver cell environment is disrupted, and the liver begins to undergo fibrotic reactions. These factors include chronic inflammation, activation of hepatic stellate cells, abnormal deposition of extracellular matrix, aberrant angiogenesis, and abnormal hepatocyte proliferation. These changes ultimately lead to alterations in the normal structure and function of the liver, resulting in hepatic fibrosis. Abbreviation: HSCs, hepatic stellate cells.

However, in response to various stimuli such as chronic liver injury, inflammation, oxidative stress (OS), infections with hepatitis viruses, and gene damage, HSCs undergo a phenotypic switch from a quiescent to an activated state, which is characterized by the loss of vitamin A-containing lipid droplets, enhanced expression of alpha-smooth muscle actin (α-SMA), increased proliferation, and secretion of large amounts of ECM proteins, particularly Col-I (Kisseleva and Brenner, 2020). The activation of HSCs is a complex process that involves multiple signaling pathways and cytokines, including transforming growth factor-β (TGF-β)1, platelet-derived growth factor (PDGF), connective tissue growth factor (CTGF), tumor necrosis factor-α (TNF-α), and interleukin (IL)-6 (Gan et al., 2022). These mediators can trigger intracellular signaling cascades that lead to the activation of downstream transcription factors such as nuclear factor kappa B (NF-κB), activator protein 1 (AP-1), and Smad proteins, which in turn regulate the expression of genes encoding ECM components, growth factors, and cytokines (Wu and Zern, 2000; Chen and Zheng, 2008; Xiang et al., 2018; Li et al., 2019; Chen et al., 2023). The accumulation of ECM proteins within the liver parenchyma is a hallmark of hepatic fibrosis (Hussein et al., 2020; Bourebaba and Marycz, 2021). The main ECM components involved in the pathogenesis of hepatic fibrosis include collagen types I (col-I)/III/IV, fibronectin, laminin, and elastin (Bauer and Habior, 2022). HSCs are the primary source of these ECM proteins in response to injury or inflammation (Ramos-Tovar and Muriel, 2020). Activated HSCs migrate from the space of Disse to the site of injury, where they proliferate and synthesize large amounts of ECM proteins, particularly col-I (Xu et al., 2020). The deposition of ECM proteins within the liver parenchyma leads to the disruption of the normal liver architecture, impairing the liver’s functions, including detoxification, metabolism, and bile secretion (Tong et al., 2022). The pathogenesis of hepatic fibrosis is shown in Figure 1.

## 3 Pathological angiogenesis and its inductive effect on hepatic fibrosis

**FIGURE 2:**
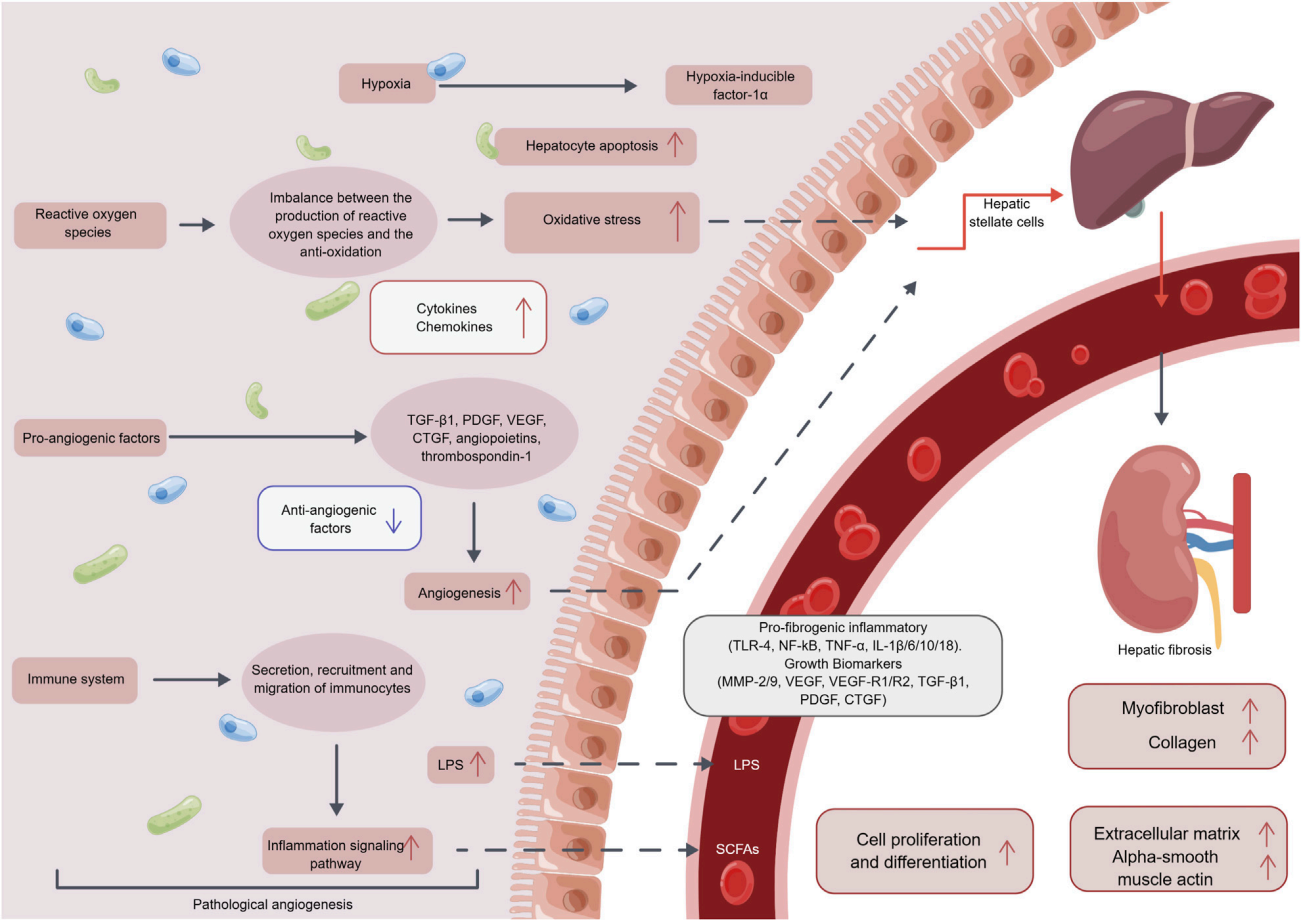
The mechanism of the pathological angiogenesis in hepatic fibrosis. After pathological angiogenesis, it can promote the secretion of inflammatory factors, chemotactic factors, and growth factors through mediating hypoxia, oxidative stress reactions, vascular endothelial growth factors, immune cells, and other pathways. This further induces pathological angiogenesis, promoting the production of myofibroblasts, collagen, extracellular matrix, alpha-smooth muscle actin, and ultimately leading to cell proliferation and differentiation, thus inducing the occurrence and development of hepatic fibrosis. Abbreviation: LPS, Lipopolysaccharide; TGF-β, transforming growth factor-β; PDGF, Platelet derived growth factor; VEGF, vascular endothelial growth factor; CTGF, Connective tissue growth factor; TLR-4, Toll-like receptor 4; NF-kB, nuclear factor kappa-B; TNF-α, tumor necrosis factor-α; IL, Interleukin; MMP, Matrix metalloproteinase; SCFAs, short chain fatty acid.

Angiogenesis is controlled by a complex interplay of pro-angiogenic and anti-angiogenic factors, including vascular endothelial growth factor (VEGF), angiopoietins, and thrombospondin-1 (TSP-1). VEGF is a potent pro-angiogenic factor that promotes the growth and survival of new blood vessels (Zhu et al., 2018; B et al., 2018). Angiopoietins are another family of pro-angiogenic factors that interact with the Tie2 receptor on endothelial cells to promote vessel sprouting and stabilization (Teichert et al., 2017). TSP-1, on the other hand, is an anti-angiogenic factor that inhibits angiogenesis by binding to CD36 receptors on endothelial cells and inducing cell death (Murphy-Ullrich and Suto, 2018). Pathological blood vessels in the liver have been shown to induce the activation of HSCs through various mechanisms, including hypoxia, OS, the secretion of growth factors and immune cells (as illustrated in Figure 2), which will eventually lead to hepatic fibrosis (Rosmorduc and Housset, 2010; Novo et al., 2012; Li et al., 2017; Li et al., 2019).

### 3.1 Hypoxia

Hypoxia itself induces a series of cellular responses that can contribute to hepatic fibrosis. One of the primary consequences of hypoxia is the activation of hypoxia-inducible factor 1 (HIF-1). HIF-1 is a transcription factor that regulates the expression of numerous genes involved in angiogenesis, inflammation, and fibrosis. Under normal conditions, HIF-1α, a subunit of HIF-1, is constantly degraded in an oxygen-dependent manner (Copple et al., 2011; Novo et al., 2012; Bahrami et al., 2018; Ruart et al., 2019; Aschner et al., 2023). However, during hypoxia, the degradation process is inhibited, leading to the accumulation of HIF-1α and subsequent activation of HIF-1 (Yang et al., 2022; Yuan et al., 2022). This activation triggers the expression of various pro-fibrotic factors, including TGF-β and CTGF (Wang Y. et al., 2013). TGF-β and CTGF plays a crucial role in hepatic fibrosis as it stimulates the activation of HSCs, the primary cells responsible for ECM production (Makino et al., 2018; Kyritsi et al., 2021). In addition to promoting fibrogenesis, hypoxia also affects tissue repair by impairing the clearance of apoptotic cells, leading to increased inflammation and tissue damage (Liu Y. et al., 2019). This chronic inflammation further perpetuates the fibrotic process in the liver (Koyama and Brenner, 2017).

Overall, pathological angiogenesis leads to hypoxia, which triggers the activation of HIF-1 and subsequent expression of pro-fibrotic factors. These factors induce the activation of HSCs, excessive ECM production, and ultimately contribute to the development of hepatic fibrosis.

### 3.2 Oxidative stress

OS contributes to the development and progression of hepatic fibrosis. OS is a state where there is an imbalance between the production of reactive oxygen species (ROS) and the ability of the body to detoxify or repair the damage caused by these species (Hybertson et al., 2011; Sies, 2015). ROS can directly activate HSCs by increasing their proliferation and migration, as well as inducing their transdifferentiation into myofibroblasts (Bernard et al., 2020). ROS can also promote the expression of genes involved in ECM synthesis, such as collagen type 1α1 (COL1A1), fibronectin, and α-SMA (Rao et al., 2019). Pathological angiogenesis can lead to OS by creating a hypoxic environment, which in turn activates signaling pathways that promote ROS production (Coulon et al., 2011). OS then triggers the activation of HSCs and disrupts the balance between ECM synthesis and degradation, ultimately leading to the development of hepatic fibrosis (Coulon et al., 2011; Novi et al., 2023).

Overall, the development of hepatic fibrosis involves a complex interplay between multiple cellular and molecular events. Pathological angiogenesis contributes to the progression of hepatic fibrosis by promoting the activation of HSCs and the production of ECM proteins. This process is accompanied by increased OS within the liver tissue, which further drives the fibrotic response. Understanding the mechanisms underlying pathological angiogenesis and OS in hepatic fibrosis may provide potential therapeutic targets for the treatment of this devastating disease.

### 3.3 Growth factors

Pathological angiogenesis, the abnormal formation of blood vessels, plays a crucial role in the development and progression of various diseases, including hepatic fibrosis. When this process occurs in the liver, it can lead to the secretion of growth factors, contributing to the fibrotic cascade (Zhang B. et al., 2021). For example, VEGF secreted by pathological blood vessels promotes the proliferation and migration of HSCs, as well as the transcriptional activation of genes involved in ECM synthesis (Arroyo and Iruela-Arispe, 2010). Similarly, the secretion of PDGF by pathological blood vessels induces the activation of HSCs and stimulates their production of ECM proteins (Yang et al., 2008). The secretion of TGF-β1 by HSCs in response to hypoxia also contributes to the activation of HSCs and the development of hepatic fibrosis (Song et al., 2019).

Ultimately, the pathological angiogenesis in hepatic fibrosis leads to the secretion of growth factors, which promote HSC activation, ECM production, and ultimately the development of fibrosis. Targeting this dysregulated angiogenesis represents a potential avenue for therapeutic intervention in the treatment of hepatic fibrosis.

### 3.4 Immune cells

Pathological blood vessels in the liver can also contribute to the pathogenesis of hepatic fibrosis through the recruitment of immune cells (Nakanuma et al., 1997; Arroyo and Iruela-Arispe, 2010). These immune cells, such as macrophages, neutrophils, and lymphocytes, are essential players in the inflammatory response. They secrete growth factors, cytokines, chemokines, and other pro-inflammatory mediators that further promote inflammation and fibrosis (Higashi et al., 2017; da Silva Meirelles et al., 2020). The immune cells recruited to the liver in response to pathological angiogenesis release growth factors like TGF-β and PDGF, leading to the accumulation of fibrosis and scar tissue in the liver (Iwakiri et al., 2014; Wilhelm et al., 2016). Additionally, the immune cells also secrete pro-inflammatory cytokines, such as TNF-α and IL-1, which contribute to the activation of HSCs and the production of ECM proteins (Bottcher and Pinzani, 2017; Ramirez-Pedraza and Fernandez, 2019). Furthermore, the immune cells recruited through pathological angiogenesis can perpetuate the inflammatory response by activating more immune cells and amplifying the secretion of pro-inflammatory cytokines, establishing a self-perpetuating cycle (Ramirez-Pedraza and Fernandez, 2019).

To sum up, pathological angiogenesis in chronic liver diseases leads to the recruitment of immune cells that secrete pro-inflammatory cytokines. This cascade of events ultimately results in the activation of HSCs, excessive deposition of ECM proteins, and the progression of hepatic fibrosis. Understanding the role of pathological angiogenesis and immune cell activation may provide potential targets for therapeutic interventions in hepatic fibrosis.

## 4 Therapeutic potential of traditional Chinese medicine monomers and single herbal extracts in inhibiting pathological angiogenesis for the treatment of hepatic fibrosis

One class of TCM compounds that has attracted interest for its potential anti-fibrotic effects is monomers, or active ingredients derived from natural herbs. Monomers are usually small molecules that can be easily isolated, purified, and studied for their pharmacological properties (Zhang Y. et al., 2020).

**FIGURE 3:**
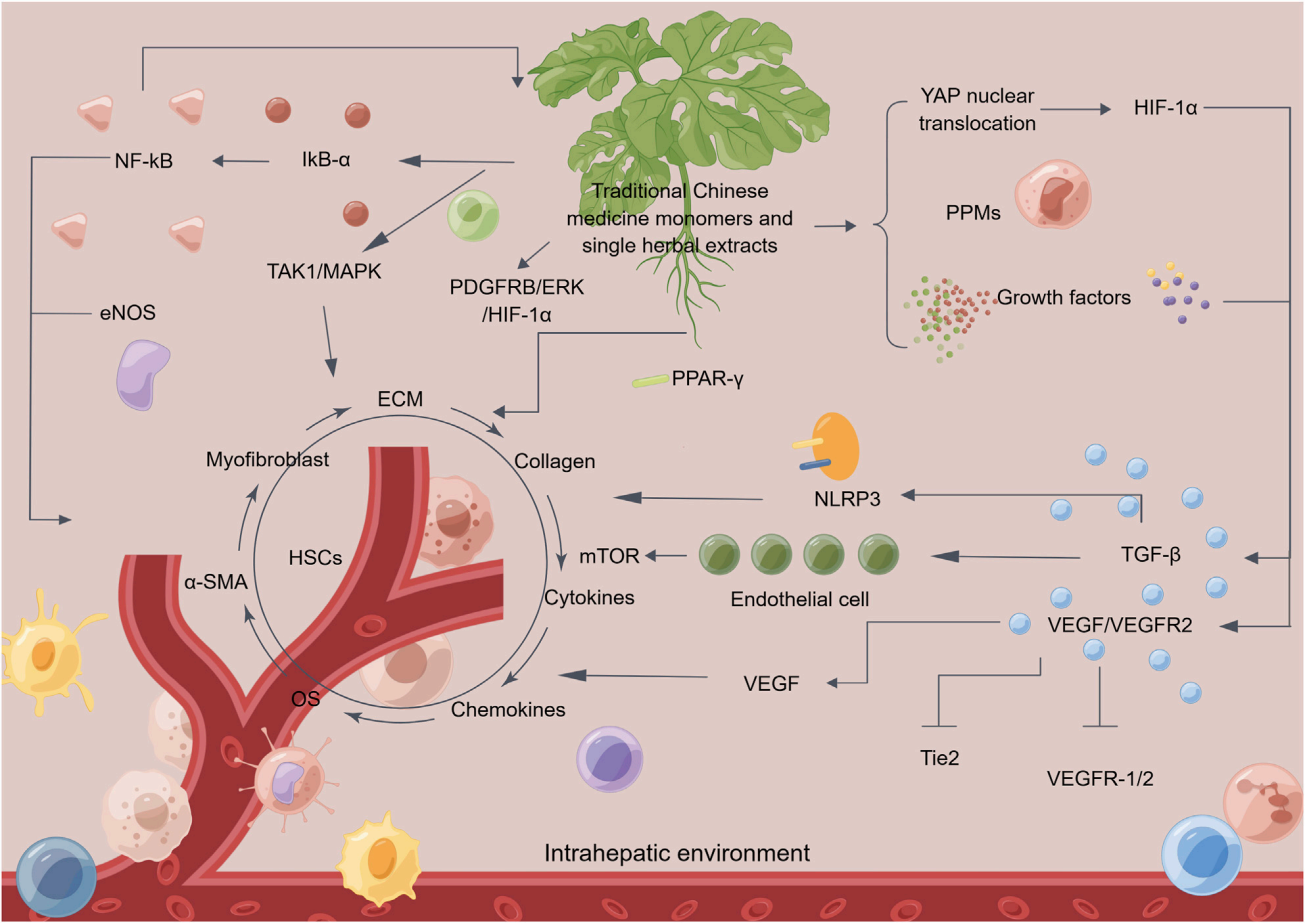
The mechanism of traditional Chinese medicine monomers and single herbal extracts in treating hepatic fibrosis by inhibiting pathological angiogenesis. Traditional Chinese medicine (TCM) monomers and single herbal extracts can reverse hepatic fibrosis in multiple ways. Firstly, it can inhibit pathological angiogenesis and regulate related signaling pathways. Additionally, it can suppress the secretion of growth factors, cytokines, and chemokines, thereby reducing angiogenesis. Moreover, TCM monomers and single herbal extracts can inhibit the activity of gene proteins related to hepatic fibrosis and oxidative stress reactions in the liver, thereby alleviating inflammation. These actions help to inhibit the secretion of fibroblasts and the activation of alpha-smooth muscle actin, reducing the deposition of collagen and extracellular matrix. TCM monomers and single herbal extracts also inhibits the activity of hepatic stellate cells, ultimately achieving the goal of reversing hepatic fibrosis. Abbreviation: HSCs, hepatic stellate cells; NF-kB, nuclear factor kappa-B; eNOS, endothelial nitric oxide synthase; IkB-α, inhibitory subunit of NF-κBα; TAK1, TGF beta-activated kinase 1; MAPK, mitogen-activated protein kinase; PDGFRB, platelet derived growth factor receptor beta; ERK, extracellular regulated kinase; HIF-1α, hypoxia-inducible factor 1α; PPAR-γ, peroxisome proliferator-activated receptor γ; YAP, yes-associated protein; PPMs, matrix metalloproteinases; TGF-β, transforming growth factor-β; VEGF, vascular endothelial growth factor; Tie2, recombinant TEK tyrosine kinase, endothelial; NLRP3, NLR family, pyrin domain containing 3; OS, oxidative stress; α-SMA, alpha-smooth muscle actin; ECM, extracellular matrix.

TCM monomers and single herbal extracts, such as curcumin, procyanidin B2, icariin, amygdalin, oroxylin A, alpinetin, salvianolic acid B, and carthami flos, among others, have been demonstrated to possess notable anti-angiogenic properties. These properties can be beneficial in inhibiting the formation of pathological angiogenesis and impeding the progression of hepatic fibrosis (Figure 3; Table 1).

**TABLE 1:** Traditional Chinese medicine monomers and single herbal extracts inhibit pathological angiogenesis to improve hepatic fibrosis.

No.	Traditional Chinese medicine	The part of the plant	Plant	Preparation	Model	Quantity of the study models	Treatment time	Up-regulating	Suppression	Mechanism	References
1	Curcumin	Root	*Curcuma longa* L.	Aqueous	Rat	30	8 weeks	PPAR-γ	α-SMA, COL1A1, fibronectin, CD34, VEGF, VEGF-R2, PDGF-βR, VEGF-R18, HIF-1α, ERK, PI4K, AKT, mTOR, PDGF, PDGF-βR/ER, PDGF-βR/FAK/RhoA, vWF, CD31, VEGFR-1, Cox-2, PDGF-βR, FAK/RhoA	The approach targets HSCs by activating PPAR-γ-dependent mechanisms, which help alleviate sinusoidal angiogenesis in hepatic fibrosis.	Zhang et al. (2014a)
2	Procyanidin B2 (PCB2)	Fruit peel	*Vaccinium* spp.	Aqueous	Mice, LX2 cell	42	4 weeks		VEGF-A, HIF-1α, α-SMA, Col-I, TGF-β1	This treatment method works by inhibiting the activation of HSCs, reducing ECM production, and inhibiting angiogenesis. Through these actions, it can reverse the progression of hepatic fibrosis both *in vivo* and *in vitro*.	Feng et al. (2019)
3	Icariin	Leaf and stem	*Epimedium brevicornu* Maxim.	Aqueous	Rat	40	4 weeks	mTOR, p70S6 kinase, BAMBI	VEGF, PDGF-β, CTGF, Ang-1, HMGB1, TGF-β, Beclin-1, TLR-4, NF-kB, IL-1β, Cox-2, TGF-β1/Smad2/CTGF	The potential mechanism of action for this treatment method involves inhibiting both angiogenesis and autophagy, which may contribute to its anti-hepatic fibrosis effect.	Algandaby et al. (2017)
4	Amygdalin	Kernel	*Prunus armeniaca* L.	Aqueous	Mice	32	3 weeks	—	Col-I, α-SMA, CD31, p-Smad2/3, TGF-β R0, TGFβ R05, p-Smad0/01, TGF-β/Smad	The treatment has been found to significantly inhibit the TGF-β/Smad signaling pathway, which effectively suppresses HSC activation. This leads to an improvement in angiogenesis and a subsequent alleviation of hepatic fibrosis.	Xiao et al. (2021)
5	Oroxylin A	Root	*Scutellaria baicalensis* Georgi	Aqueous	Mice	30	4 weeks	—	VEGF-A, Ang-2, PECAM-1/CD31, HIF-1α, YAP	This treatment method works by inhibiting hypoxia-induced YAP nuclear translocation, leading to a reduction in the transcription of downstream target genes, such as VEGF-A and Ang-2. This ultimately results in anti-angiogenic activity, which helps in preventing and treating hepatic fibrosis.	Zhang et al. (2018)
6	Alpinetin	Seed	*Alpinia katsumadai* Hayata	Infusion	Mice	40	4 weeks	—	TNF-α, IL-1β/6/10/18, IFN-γ, VEGF, PDGF, 70-dichlorofluorescin, α-SMA, fibronetin, α1(I) procollagen, Cox-2, iNOS, VEGF-R2, PDGF-βR, HIF-1α, NLRP3, Caspase-1 p20, ASC, HO-1, NQO1, GCLM, GCLC	The treatment method works by inhibiting the NLRP3-mediated anti-inflammatory activity and Nrf2-mediated antioxidant activity, leading to a reduction in liver angiogenesis and an overall improvement in hepatic fibrosis.	Zhu et al. (2021a)
7	Salvianolic Acid B (SalB)	Root	*Salvia miltiorrhiza* Bge.	Infusion	Rat	48	6 weeks	NF-kB and IkB-α protein in the cytoplasm	HA, IV-C, LN, PIIIP, NF-kB in nucleolus	The mechanism of action involves the regulation of NF-kB and IkB-α expression, which is responsible for halting the progression of liver angiogenesis and alleviating hepatic fibrosis.	Wang et al. (2012)
8	Carthami flos	Flower	*Carthamus tinctorius* L.	Aqueous	Mice	36	6 weeks	—	HYP, Col-IV, LN, HA, PC-III, PDGFRB, p-MEK, p-ERK1/2, HIF-1α, VEGF-A, VEGF-R2, AKT, eNOS, α-SMA, Col-I, CD31/34, vWF, PDGFRB/ERK/HIF-1α, VEGFA/AKT/eNOS	Resisting angiogenesis in hepatic fibrosis through the PDGFRB/ERK/HIF-1α and VEGFA/AKT/eNOS signaling pathways may serve as a promising therapeutic approach for treating hepatic fibrosis.	Xue et al. (2023)

### 4.1 Curcumin

Curcumin is a natural polyphenolic compound found in *Curcuma longa* L., a spice commonly used in traditional medicine for centuries (Anand et al., 2007). Curcumin has been shown to possess anti-inflammatory, antioxidant, and anticancer properties, making it a promising therapeutic candidate for hepatic fibrosis (Zia et al., 2021). Recent studies have suggested that curcumin may exert its beneficial effects on hepatic fibrosis by inhibiting angiogenesis. Studies have shown that curcumin can modulate several key signaling pathways involved in angiogenesis, including the VEGF and angiopoietin/Tie2 pathways (Kityania et al., 2023). Curcumin has been shown to reduce the expression of VEGF and its receptor, VEGFR-2, in hepatic fibrosis models (Gimenez-Bastida et al., 2022). In addition, curcumin has been shown to inhibit the activation of the Tie2 receptor by angiopoietins, leading to a reduction in vessel sprouting and stabilization (Kityania et al., 2023). A study suggests that curcumin can inhibit and destroy VEGF expression in HSCs by targeting the PDGF-Rβ/extracellular regulated protein kinases (ERK) and mTOR pathways, as well as blocking the PDGF-Rβ/adhesion kinase/Ras homolog gene family, member A (RhoA) cascade reaction (Zhang F. et al., 2014). This indicates that curcumin may be effective in treating sinusoidal angiogenesis and have anti-fibrotic properties by activating PPAR-γ-dependent mechanisms (Zhang F. et al., 2014). Furthermore, curcumin has been shown to improve carbon tetrachloride-induced angiogenesis and sinusoidal capillary formation by inhibiting various pro-angiogenic factors, leading to an improvement in hepatic fibrosis (Yao et al., 2013). All in all, the relationship between pathological blood vessels and curcumin in hepatic fibrosis represents a promising avenue for the prevention and treatment of this chronic liver disease. Curcumin’s ability to inhibit angiogenesis through modulation of key signaling pathways, such as the VEGF, angiopoietin/Tie2, PDGF-Rβ/ERK and mTOR pathways, provide a rationale for its clinical development. However, further studies are needed to fully elucidate the mechanisms underlying its effects and to determine its clinical potential.

### 4.2 Procyanidin B2

Procyanidin B2 (PCB2) is a natural flavonoid compound of *Vaccinium* spp. that has been shown to have anti-inflammatory, anti-oxidant, and anti-cancer properties (Zhao J. et al., 2022). Recent studies have also suggested that PCB2 may have a beneficial effect on hepatic fibrosis by targeting pathological blood vessels in the liver. One study has shown that PB2 suppressed both cell vitality and function, including invasion, migration, and epithelial-mesenchymal transition (EMT) (Sun et al., 2022). Additionally, it inhibited the VEGF/VEGFR2 pathway, which resulted in reduced cell growth and angiogenesis (Sun et al., 2022). Additionally, another study found that PCB2 was effective in treating hepatic fibrosis, as it was tested on two different systems: a carbon tetrachloride (CCl4)-induced mouse model and a human HSC line known as LX2 cells (Feng et al., 2019). The results of these tests showed that PCB2 inhibited the growth of HSCs and induced apoptosis or programmed cell death (Feng et al., 2019). Additionally, it decreases the levels of multiple molecules associated with pathological angiogenesis in hepatic fibrosis under both *in vivo* and *in vitro* conditions. These molecules include VEGF-A, HIF-1α, α-SMA, Col-1, and TGF-β1 ^71^. Further testing revealed that PCB2 targets the Hedgehog (Hh) pathway which plays a crucial role in hepatic fibrosis. PCB2 inhibited the Hh pathway and therefore reduced the activation, production of ECM, and angiogenesis of HSCs (Feng et al., 2019). As a consequence, it was able to reverse the progression of hepatic fibrosis in both *in vivo* and *in vitro* situations (Feng et al., 2019). On the whole, these studies suggest that PCB2 may have significant therapeutic potential for the treatment of hepatic fibrosis and other related conditions. Further research is needed to fully understand its mechanisms of action in human clinical trials. However, these early findings provide an important step towards the development of new, effective treatments for these diseases.

### 4.3 Icariin

Icariin is a flavonoid compound extracted from the Chinese herb *Epimedium brevicornu* Maxim (Hong et al., 2022). It has been reported to have various pharmacological properties, including anti-inflammatory, antioxidant, and anti-fibrotic effects (Szabo et al., 2022). Recent studies have shown that icariin may have therapeutic potential for the treatment of hepatic fibrosis by targeting pathological blood vessels in the liver. Several studies have investigated the effect of icariin on pathological blood vessels in the liver. For example, one study showed that icariin can inhibit angiogenesis, the process by which new blood vessels are formed, in a mouse model of hepatic fibrosis (Li et al., 2022). The researchers found that icariin reduced the expression of VEGF, a key regulator of angiogenesis, and its receptor VEGFR-2, in the liver tissue (Li et al., 2022). This led to a reduction in the number of blood vessels One possible mechanism is through the regulation of the TGF-β signaling pathway, which playand a decrease in hepatic fibrosis (Li et al., 2022). Another study investigated the effect of icariin on hepatic fibrosis. s a key role in the development of hepatic fibrosis. Studies have shown that icariin can inhibit the expression of TGF-β and its downstream signaling molecules in the liver, leading to a reduction in collagen deposition and hepatic fibrosis (Wang et al., 2021). To sum up, icariin is a natural compound with potential therapeutic benefits for the treatment of hepatic fibrosis. Its effects on pathological blood vessels in the liver, make it a promising candidate for the development of novel anti-fibrotic therapies. Further studies are needed to fully elucidate the mechanisms underlying its beneficial effects and to determine its efficacy and safety in humans.

### 4.4 Amygdalin

Amygdalin, also known as vitamin B17, is a natural compound found in the kernels of *Prunus armeniaca* L. (He et al., 2020). It has been used in traditional medicine for the treatment of cancer and other diseases, but its effects on hepatic fibrosis have not been extensively studied. Recent research has suggested that amygdalin may have beneficial effects on pathological blood vessels in the liver, making it a potentially promising therapy for hepatic fibrosis (Cao and Deng, 2016). In animal models of hepatic fibrosis, amygdalin has been shown to inhibit the expression of pro-angiogenic factors such as VEGF and inhibit the proliferation of endothelial cells, which are the cells that line blood vessels (Attia et al., 2022). Moreover, amygdalin has been demonstrated to have a significant ability to alleviate carbon tetrachloride-induced hepatic fibrosis, as well as inhibit the expression of col-I, α-SMA, CD1, and the TGF-β/Smad signaling pathway (Xiao et al., 2023). These effects lead to the suppression of HSCs activation and a subsequent improvement in vascular formation (Xiao et al., 2023). This makes it a potentially effective drug for treating hepatic fibrosis (Xiao et al., 2023). Despite these promising findings, more research is needed to fully elucidate the mechanisms underlying amygdalin’s effects on pathological blood vessels in the liver.

### 4.5 Oroxylin A

Oroxylin A is a natural compound derived from the roots of the Chinese herb *Scutellaria baicalensis* Georgi (Yao et al., 2022). It has been shown to have potent anti-angiogenic and anti-inflammatory effects, making it a promising candidate for the treatment of hepatic fibrosis. Oroxylin A exhibits anti-angiogenic activity by inhibiting hypoxia-induced YAP nuclear translocation (Zhang et al., 2018). This inhibitory effect may lead to reduced accumulation of HIF-1α and transcription of downstream target genes, such as VEGF-A and Ang-2 (Zhang et al., 2018). Consequently, it helps to protect liver function and prevent liver tissue damage, thus achieving the goal of preventing and treating hepatic fibrosis (Zhang et al., 2018). Another study investigated the effects of oroxylin A on HSC activation and angiogenesis *in vitro* (Zhang L. et al., 2021). The researchers found that oroxylin A inhibited the activation of HSCs by reducing their expression of α-SMA and col-I (Chen et al., 2018). In addition, oroxylin A also inhibited the growth and migration of endothelial cells, indicating its potent anti-angiogenic effects (Gao et al., 2010). In short, these studies suggest that oroxylin A has significant potential as a therapeutic option for the treatment of hepatic fibrosis. By targeting pathological blood vessels and ameliorating hypoxia, oroxylin A can slow the progression of fibrosis and promote the regeneration of damaged liver tissue.

### 4.6 Alpinetin

Alpinetin, a natural flavonoid compound of *Alpinia katsumadai* Hayata, has been shown to inhibit angiogenesis and exert anti-fibrotic effects in hepatic fibrosis (Zhao G. et al., 2022). One study investigated the potential mechanisms underlying the anti-angiogenic effects of alpinetin in hepatic fibrosis (Zhu Z. et al., 2021). The study found that the effects of alpinetin which has been shown to decrease angiogenesis in the liver of experimental animals with fibrosis, hinder anti-inflammatory activity mediated by NOD-like receptor thermal protein domain associated protein 3 (NLRP3), impede antioxidant activity mediated by nuclear factor erythroid 2-related factor 2 (Nrf2), and have a positive therapeutic effect on hepatic fibrosis (Zhu Z. et al., 2021). In conclusion, alpinetin has been shown to inhibit angiogenesis and exert anti-fibrotic effects in hepatic fibrosis by inhibiting NLRP3 and Nrf2 signaling. These findings suggest that alpinetin may be a promising therapeutic agent for the treatment of hepatic fibrosis and other angiogenesis-related diseases. Therefore, further in-depth studies are required to explore the full potential of alpinetin in treating hepatic fibrosis and related conditions.

### 4.7 Salvianolic acid B

Salvianolic Acid B (SalB) is a naturally occurring polyphenol derived from the Chinese herb *Salvia miltiorrhiza* Bge. (Chen J. et al., 2022). Over the years, there has been extensive research on the therapeutic potential of SalB due to its strong antioxidant, anti-inflammatory, and anti-fibrotic effects (Zou et al., 2022). Researchers have found that SalB can significantly inhibit the growth of pathological blood vessels in the liver and prevent the progression of hepatic fibrosis (Zhao X. S. et al., 2019). Recent studies have shown that SalB can inhibit the growth of pathological blood vessels by modulating several key signaling pathways involved in angiogenesis. For example, SalB has been shown to block the expression of VEGF, thereby preventing the formation of new blood vessels (Lay et al., 2003). SalB has also been shown to modulate the expression of matrix metalloproteinases (MMPs), enzymes that break down the ECM and promote angiogenesis (Lv et al., 2010). Furthermore, SalB has been shown to inhibit the activation of HSCs through several mechanisms, including the inhibition of TGF-β1 signaling (Lin et al., 2006). In an experiment exploring the protective effect of SalB on hepatic fibrosis, it was found that SalB can inhibit the progression of liver angiogenesis and alleviate hepatic fibrosis by regulating the expression of NF-kB and inhibitor of NF-kB, IkB-α (Wang et al., 2012). In conclusion, the relationship between pathological blood vessels and SalB in the context of hepatic fibrosis is complex and multifaceted. On the one hand, pathological blood vessels are a result of the inflammatory response triggered by liver injury, and SalB is known to have potent anti-inflammatory effects. On the other hand, pathological blood vessels contribute to the development and maintenance of hepatic fibrosis by providing a source of nutrients and oxygen to the fibrotic tissue, and SalB has been shown to inhibit their growth by modulating several key signaling pathways involved in angiogenesis. Taken together, these findings suggest that SalB may be a promising therapeutic agent for the treatment of hepatic fibrosis.

### 4.8 Carthami flos

Carthami flos, found in *Carthamus tinctorius* L., is an herb that has long been used in TCM to treat liver diseases (Ao et al., 2018). It is known for its anti-inflammatory, antioxidant, and antifibrotic properties, which make it a promising candidate for treating hepatic fibrosis (Ye et al., 2021). Carthami flos contains compounds like flavonoids, anthocyanins, and carthamin, which have been shown to inhibit the activity of inflammatory cells and reduce the production of ECM (Nobakht et al., 2000; Han et al., 2017). In 2023, it was discovered that carthami flos has the potential to reduce elevated levels of liver enzymes such as alanine transaminase (ALT), aspartate aminotransferase (AST), alkaline phosphatase (ALP), and glutamyl transferase (γ-GT) (Satoh et al., 2004). In addition to this, it can also lower the levels of hydroxyproline (HYP), col-III/IV and laminin associated with hepatic fibrosis (Xue et al., 2023). Carthami flos can prevent the upregulation of several proteins involved in fibrosis including PDGF-Rβ, phosphorylated MEK kinase, p-ERK1/2, HIF-1α, VEGF-A, VEGF-R2, protein kinase B (AKT), and endothelial nitric oxide synthase (eNOS), induced by carbon tetrachloride and gradual disappearance of sinusoidal capillaries (Xue et al., 2023). Moreover, the levels of platelet endothelial cell adhesion molecule-1 (PECAM-1), CD31, CD34, and vascular pseudo-hemophilia factor were decreased, while an increase in sinusoidal endothelial cell counts was observed, indicating improved vascular health (Xue et al., 2023). These findings suggest that carthami flos may have potential therapeutic benefits for hepatic fibrosis and related disorders. Further studies are needed to identify the active components responsible for these effects. Nonetheless, this discovery opens up new possibilities for the development of novel treatments for liver diseases using carthami flos.

Hepatic fibrosis is a complex disease characterized by the progressive accumulation of ECM and the formation of scar tissue, leading to organ dysfunction and ultimately, cirrhosis and liver failure. Pathological angiogenesis has emerged as a key driver of hepatic fibrosis, promoting the proliferation and migration of endothelial cells and the formation of new blood vessels. TCM monomers and single herbal extracts offer potential therapeutic benefits for the treatment of hepatic fibrosis by targeting pathological angiogenesis which can mediate the hypoxia, inflammation, OS and growth factors. While further research is needed to fully elucidate their mechanisms of action and clinical efficacy in humans, TCM monomers and single herbal extracts represent a promising avenue for the development of novel therapeutics for the treatment of hepatic fibrosis.

## 5 Treatment of hepatic fibrosis by traditional Chinese medicine formulations targeting pathological angiogenesis

**FIGURE 4:**
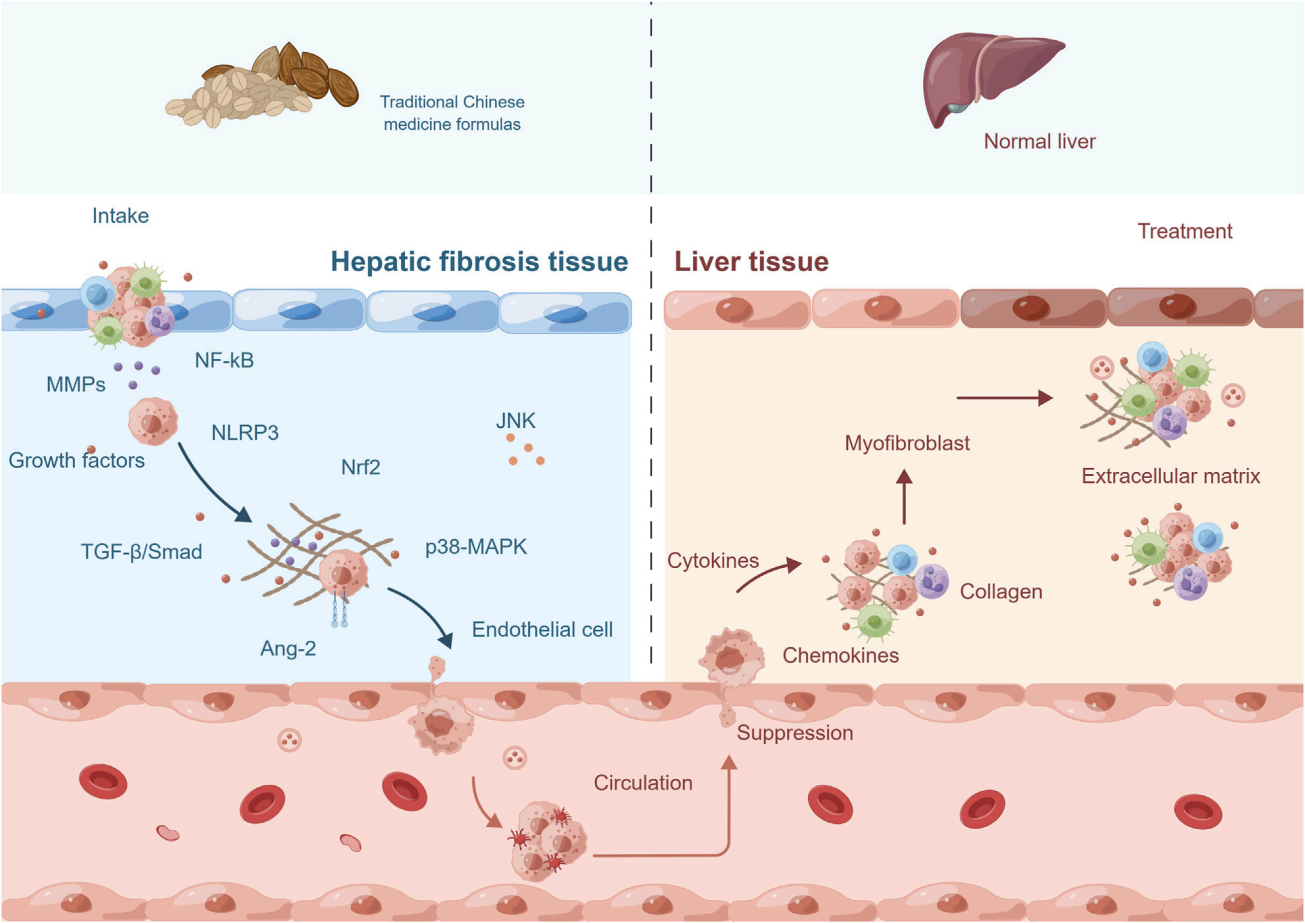
The mechanism of traditional Chinese medicine formulas in treating hepatic fibrosis by inhibiting pathological angiogenesis. The mechanism of action of traditional Chinese medicine (TCM) formulas in treating hepatic fibrosis involves multiple aspects. Firstly, they can suppress the pathological angiogenesis and the activity of related signaling pathways. Secondly, they exert therapeutic effects by downregulating the expression levels of relevant gene proteins. Additionally, they can inhibit the secretion of growth factors, chemokines, and cytokines, thus blocking the progression of fibrosis. Moreover, TCM formulas can also suppress the activity of fibroblasts to prevent excessive proliferation. Lastly, they can promote the degradation of collagen and extracellular matrix, helping to restore the normal structure and function of the liver. These comprehensive actions constitute important mechanisms through which TCM formulas exert therapeutic effects in the treatment of hepatic fibrosis. Abbreviation: NF-kB, nuclear factor kappa-B; MMPs, matrix metalloproteinases; NLRP3, NLR family, pyrin domain containing 3; Nrf2, nuclear factor erythroid-2-related factor 2; TGF-β, transforming growth factor-β; Ang-2, angiopoietins-2; MAPK, mitogen-activated protein kinase; JNK, c-Jun n-terminal kinase.

TCM has a long history of using complex herbal formulas to treat various diseases, including hepatic fibrosis. The use of TCM formulas for the treatment of hepatic fibrosis is based on the concept of treating the root cause of the disease, rather than just the symptoms. These formulas are designed to target multiple pathological mechanisms underlying hepatic fibrosis, including inflammation, OS, and angiogenesis. The mechanism of TCM formulas (for example, Fuzheng Huayu Formula, Xiayuxue Decoction, Xuefuzhuyu decoction and Ger-Gen-Chyn-Lian-Tang) in treating hepatic fibrosis by inhibiting pathological angiogenesis may involve multiple biological pathways and molecular signals (Figure 4; Table 2). Some of the active ingredients of TCM formulas can inhibit hematopoietic stem cell-hematopoietic cell pathway, the expression of VEGF and VEGFR, TGF-β, inflammatory mediators, and other pathways to inhibit the process of pathological angiogenesis and reduce hepatic fibrosis.

**TABLE 2:** Traditional Chinese medicine formulas inhibit pathological angiogenesis to improve hepatic fibrosis.

No.	Traditional Chinese medicine	Component	Efficacy	Preparation	Model	Quantity of the study models	Treatment time	Up-regulating	Suppression	Mechanism	References
1	Fuzheng Huayu Formula	Salvia miltiorrhiza 4 g, Peach Kernel 2 g, fermented Cordyceps sinensis 8 g, Pine Pollen 2 g, Bugleweed 6 g, Schisandra chinensis 2 g	Promoting blood circulation and removing blood stasis, nourishing essence and liver	Aqueous	Mice	48	3 weeks		α-SMA, Col-I, HYP, the number and diameters of fenestra of LSEC, HIF-1α, VEGF, VEGF-R2/R4, p-ERK1/2	By improving vascular remodeling, it can contribute to the improvement of hepatic fibrosis.	Liu et al. (2019b)
2	Xiayuxue Decoction	Rhubarb 9 g, Peach Kernel 9 g, Silkworm 9 g	Promoting blood circulation and removing blood stasis	Aqueous	Mice	-	3 weeks	Bax, cyt-c, caspase-2/3	JNK, p38-MAPKs,α-SMA, Col-1, Bcl-1/2, CD31, VEGF	By inhibiting the expression of JNK and p38-MAPKs and reducing angiogenesis, it can protect liver function and tissue, as well as improve hepatic fibrosis.	Zhang et al. (2020b)
3	Xuefuzhuyu Decoction (XFZYD)	Peach Kernel 12 g, Carthamus tinctorius 9 g, Angelica sinensis 9 g, Rehmannia glutinosa 9 g, Achyranthes bidentata 9 g, Ligusticum chuanxiong 4.5 g, Platycodon grandiflorus 4.5 g, Paeonia lactiflora 6 g, Aurantium 6 g, Glycyrrhiza 6 g, Bupleurum 3 g	Promoting blood circulation, removing blood stasis, promoting qi circulation and relieving pain	Aqueous	Mice	49	3 weeks	DDAH1	α-SMA, Col-I, CD31, VEGF, VEGF-R2, HIF-1α, ADMA	In addition to suppressing collagen deposition, it also has an anti-angiogenic effect on the fibrotic liver, which helps to inhibit the progression of hepatic fibrosis.	Zhou et al. (2014)
4	Ger-Gen-Chyn-Lian-Tang (GGCLT)	Kudzu Root 15 g, Coptis chinensis 9 g, Glycyrrhiza 6 g, Scutellaria baicalensis 9 g	Turn over the exterior and clear the interior	Aqueous	Mice	38	4 weeks		HYP, HIF-1α, TGF-β, MMP-2/9, VEGF, VEGF-R1/R2, Col-III	It prevents or reduces hepatic fibrosis by inhibiting the occurrence of OS and angiogenesis in the liver.	Chang et al. (2019)

-, the reference does not explain.

### 5.1 Fuzheng Huayu Formula

Fuzheng Huayu Formula is a TCM that has been used for many years to treat liver disease. It is a combination of multiple herbs and has been shown to have anti-fibrotic effects in hepatic fibrosis. Fuzheng Huayu Formula has been found to improve liver function, reduce inflammation, and promote the regeneration of liver cells. Recent studies suggest that Fuzheng Huayu Formula can also regulate pathological blood vessels in hepatic fibrosis. Fuzheng Huayu Formula has been found to inhibit angiogenesis in the liver by reducing the expression of pro-angiogenic factors, such as VEGF and PDGF (Chen et al., 2020). Additionally, Fuzheng Huayu Formula can promote the expression of MMPs (Xing et al., 2022), which are enzymes that promote the degradation of ECM and are involved in the development of pathological blood vessels (Rosenberg, 2017). In a study conducted on rats, hepatic fibrosis was induced by injecting them with CCl4 (Liu H. L. et al., 2019). These rats were then treated with Fuzheng Huayu Formula for 6 weeks (Liu H. L. et al., 2019). The results demonstrated that the herbal formula significantly reduced the number of new blood vessels and microvessel density in their livers (Liu H. L. et al., 2019). The mechanism by which Fuzheng Huayu Formula regulates liver sinusoidal endothelial cells (LSECs) in hepatic fibrosis was also studied (Liu H. L. et al., 2019). LSECs are a crucial type of endothelial cells in the liver, which play a vital role in the development of hepatic fibrosis (Chen T. et al., 2022). The study also showed that Fuzheng Huayu Formula can decrease the production of ROS in LSECs and increase the expression of eNOS. This resulted in the improvement of endothelial function and the inhibition of angiogenesis in the liver (Liu H. L. et al., 2019). To put it briefly, Fuzheng Huayu Formula has been shown to have anti-angiogenic effects in hepatic fibrosis through its regulation of VEGF, PDGF, and MMP-2 expression, as well as its ability to reduce ROS production and enhance eNOS expression in LSECs. These findings suggest that Fuzheng Huayu Formula may be a promising therapeutic agent for the treatment of hepatic fibrosis by targeting pathological blood vessels.

### 5.2 Xiayuxue Decoction

Xiayuxue Decoction is a Chinese herbal formula that has been used for centuries to treat hepatic fibrosis (Li, 2020). With its rich history of use, there has been significant interest in investigating the efficacy and mechanisms of Xiayuxue Decoction, particularly in relation to the treatment of hepatic fibrosis. Studies have demonstrated that Xiayuxue Decoction can improve vascular remodeling in hepatic fibrosis by down-regulating the expression of certain proteins, such as JNK and p38-MAPKs, which are involved in regulating angiogenesis and vascular remodeling (Zhang D. et al., 2020). By modulating these proteins, Xiayuxue Decoction can promote the formation of stable sinusoidal capillaries and restore normal blood flow to the liver (Zhang D. et al., 2020). Inhibition of angiogenesis, or the formation of new blood vessels, is another critical mechanism by which Xiayuxue Decoction can target pathological blood vessels in hepatic fibrosis. Studies have shown that Xiayuxue Decoction can inhibit angiogenesis by reducing the expression of α-SMA and CD31, which are essential in promoting angiogenesis (Zhang L. J. et al., 2014). By down-regulating these proteins, Xiayuxue Decoction can reduce the formation of new blood vessels, thereby limiting the progression of hepatic fibrosis (Zhang L. J. et al., 2014). Moreover, Xiayuxue Decoction can significantly reduce the activity of MMP-0/9, as well as the expression of CD2, von Willebrand factor (vWF), VEGF, VEGF-R2, complement decay-accelerating factor, and α-SMA, thereby improving hepatic fibrosis (Du et al., 2011). In essence, Xiayuxue Decoction, a Chinese herbal formula with a long history of use, has been shown to modulate pathological blood vessels in hepatic fibrosis through several mechanisms, including improving vascular remodeling and inhibiting angiogenesis. These effects have been demonstrated *in vitro* and *in vivo*, and suggest that Xiayuxue Decoction may serve as an effective treatment for hepatic fibrosis. However, further studies are needed to fully elucidate the mechanisms of action of Xiayuxue Decoction and to determine its optimal clinical use.

### 5.3 Xuefuzhuyu decoction

Xuefuzhuyu decoction (XFZYD) is a traditional Chinese herbal formula that has been used for centuries to treat various medical conditions, including hepatic fibrosis (Shi et al., 2017). According to TCM theory, XFZYD works by promoting the flow of blood and regulating the balance of yin and yang in the body (Zhao Y. et al., 2019). In recent years, several studies have investigated the effects of XFZYD on pathological blood vessels in hepatic fibrosis, with promising results. One study published in 2014 by Zhou et al. (1) investigated the effects of XFZYD on angiogenesis in the liver of mices with CCl4 -induced fibrosis. The researchers found that treatment with XFZYD significantly reduced the number of pathological blood vessels in the fibrotic liver, as well as the expression of VEGF and VEGFR-2 (Zhou et al., 2014). XFZYD also inhibited the activation of HSCs and the deposition of collagen in the liver, indicating a potential anti-fibrotic effect (Zhou et al., 2014). To sum up, pathological blood vessels are a critical component of hepatic fibrosis, and targeting angiogenesis represents a promising therapeutic strategy. XFZYD, a traditional Chinese herbal formula, has been shown to have potent anti-angiogenic effects in preclinical models of hepatic fibrosis, as well as to improve the microcirculation of blood in the liver. Further research is needed to elucidate the underlying mechanisms of these effects and to evaluate the potential clinical utility of XFZYD in the treatment of hepatic fibrosis.

### 5.4 Ger-Gen-Chyn-Lian-Tang

Ger-Gen-Chyn-Lian-Tang (GGCLT), a traditional Chinese herbal formula, has been used for centuries to treat a wide range of diseases, including hepatic fibrosis (Yang, 2014). GGCLT contains several active compounds, including Kudzu Root 15 g, Coptis chinensis 9 g, Glycyrrhiza 6 g, Scutellaria baicalensis 9 g (Ho et al., 2012). These compounds have been shown to have anti-inflammatory, antioxidant, and anti-angiogenic effects, making GGCLT an attractive candidate for the treatment of hepatic fibrosis (Ho et al., 2012). The anti-angiogenic effects of GGCLT have been demonstrated in preclinical studies. In one study, GGCLT inhibited expression of VEGF, VEGFR-1/2, MMP-2/9, TGF-β factors that promote angiogenesis. This suggests that GGCLT may inhibit angiogenesis by suppressing the expression of these pro-angiogenic factors. Another study found that GGCLT can also promote the regression of existing blood vessels. In this study, GGCLT’s antioxidant properties are likely achieved through the regulation of the TGF-β/TGF-β receptor pathway by down-regulating signaling pathways such as α-SMA and lipid peroxidation (Chang et al., 2014). TGF-β plays a critical role in angiogenesis as it promotes endothelial cell proliferation and migration while increasing their sensitivity to external stimuli (Wang X. et al., 2013). Moreover, TGF-β induces the expression of matrix metalloproteinases and other proteases that degrade the basement membrane, creating a favorable environment for angiogenesis to occur (Zhu L. et al., 2021). In brief, GGCLT shows great potential as a natural therapeutic agent for the treatment of hepatic fibrosis by regulating pathological blood vessels. Through its anti-angiogenic, anti-inflammatory, and antioxidant properties, GGCLT may be able to improve liver function, reverse fibrosis progression, and prevent liver failure. More research is needed to fully understand the underlying mechanisms of action of GGCLT and to determine its clinical efficacy in the treatment of hepatic fibrosis.

In short, TCM has a long history of treating hepatic fibrosis and other liver diseases. The above-mentioned prescriptions have been found to effectively inhibit pathological angiogenesis and reduce hepatic fibrosis by regulating various signaling pathways and improving liver function. However, the use of TCM should be under the guidance of a qualified practitioner and combined with Western medicine if necessary. In addition, more rigorous clinical trials are needed to confirm the efficacy and safety of TCM in treating hepatic fibrosis.

## 6 Conclusion and discussion

Hepatic fibrosis is a condition in which there is an accumulation of excess scar tissue in the liver. This scarring can occur due to a variety of factors, including chronic liver disease, alcohol abuse, hepatitis infections, and other conditions that cause damage to the liver. As hepatic fibrosis progresses, it can lead to cirrhosis, a more severe form of liver disease that can result in liver failure and even death. Therefore, it is important to detect and treat hepatic fibrosis at an early stage before it progresses to more severe stages. Current treatments for hepatic fibrosis include medications that can slow down or stop the progression of fibrosis, as well as lifestyle modifications such as avoiding alcohol, losing weight, and maintaining a healthy diet. However, these treatments are not always effective, and there are limitations to their use. For example, some medications can have significant side effects, and they do not always address the underlying causes of hepatic fibrosis. Moreover, lifestyle modifications may not be sufficient for patients with advanced hepatic fibrosis or cirrhosis. Therefore, this article reviews the effects of TCM monomers, single herbal extracts and TCM formulas on inhibiting pathological angiogenesis, improving hepatic hypoxia, regulating cytokines, chemokines, and growth factors secretion. Additionally, they can effectively inhibit OS and inflammation, thus achieving the goal of reversing hepatic fibrosis. By providing these research findings, this article aims to provide scientific research ideas and inspirations for future development.

TCM monomers are derived from one herb or plant, which means they contain specific compounds that target specific areas of the body. This targeted approach is important in treating hepatic fibrosis because it allows for the specific targeting of liver cells and the reduction of pathological angiogenesis, inflammation and fibrosis in the liver. TCM uses TCM monomers and single herbal extracts to treat hepatic fibrosis with great success. TCM monomers and single herbal extracts are natural remedies that do not require the use of synthetic chemicals or medications. This makes them a safer alternative to conventional treatment options that can have harmful side effects. TCM monomers and single herbal extracts can be used in conjunction with Western medicine treatments, providing a holistic approach towards hepatic fibrosis treatment. In conclusion, TCM monomers and single herbal extracts offer several advantages in treating hepatic fibrosis including targeted therapy, natural remedies, fewer side effects, and integration with Western medicine treatments.

TCM formulas are made from natural substances such as herbs, minerals, and animal products, and are generally safe with minimal side effects when prescribed by a qualified practitioner. They are administered in small doses over a period of time, allowing the body to adjust to the treatment. TCM formulas are tailored to the individual’s specific needs and symptoms. Each person with hepatic fibrosis may experience different symptoms and underlying imbalances, such as liver Qi stagnation, Damp-Heat, Blood stasis, etc. TCM practitioners will prescribe a unique combination of herbs and formulas based on the patient’s diagnosis, taking into account their constitution, age, and overall health. TCM formulas can be used alone or in combination with Western medicine to provide a more comprehensive treatment. TCM formulas can help alleviate symptoms and improve liver function while minimizing the side effects of Western drugs. Studies have shown that TCM formulas (such as Fuzheng Huayu Formula, Xiayuxue Decoction, XFZYD and GGCLT) can inhibit pathological angiogenesis, reduce inflammation, improve hypoxia and liver function, and slow the progression of hepatic fibrosis.

However, from the perspective of TCM theory and practice, there are certain limitations in the application of TCM’s basic principles and treatments. Firstly, TCM emphasizes the principle of “differentiation and treatment,” which means individualized treatment based on the specific condition and constitution of the patient. However, in real clinical practice, due to limitations in time and resources, doctors may not be able to perform detailed differentiation and treatment, resulting in treatment outcomes that may not meet expectations. This is because differentiation and treatment require detailed inquiry into the patient’s medical history, observation, pulse diagnosis, tongue diagnosis, etc., which require a significant amount of time and energy. However, in a busy clinical setting, doctors may not be able to allocate enough time and attention to each patient, which limits the application of differentiation and treatment.

In addition, although TCM has a long history and rich experience, its theoretical system and treatment methods differ from modern medicine. Due to a lack of scientific evidence, TCM may not achieve clear therapeutic effects in the treatment of certain diseases, or its effectiveness may be limited. With the development of modern medicine, the diagnosis and treatment of certain diseases have become more scientific and precise. The differences between TCM’s theoretical system and treatment methods compared to modern medicine limit its effectiveness in the treatment of certain specific diseases. For example, in the fields of surgical treatment and cancer treatment, modern medicine has made significant progress, while the application of TCM in these areas is relatively limited, highlighting the limitations of TCM.

In the future, TCM can overcome these limitations and make progress through the following aspects of development. Firstly, strengthening scientific research on TCM theories and diagnostic methods, including the study of TCM (TCM monomers, single herbal extracts and TCM formulas) and the mechanism of action of TCM on diseases. This will provide more scientific basis and evidence for TCM, enhancing its reliability and effectiveness in clinical treatment. Secondly, TCM can complement and integrate with modern medicine, leveraging the strengths of both. For example, TCM’s differentiation and treatment can be combined with Western medicine’s pathological diagnosis and drug treatment to form comprehensive treatment plans. At the same time, TCM can learn from advanced technologies and equipment in modern medicine to improve diagnostic and treatment levels. Thirdly, the current research on TCM monomers, single herbal extracts and TCM formulas is mainly focused on animal *in vivo* and *in vitro* studies, with limited clinical research. Therefore, in the future, it is necessary to strengthen clinical research to validate the efficacy and safety of these TCM. Furthermore, it is crucial to further explore their mechanisms of action and clarify their relationship with the development of hepatic fibrosis. Additionally, the development of new TCM formulas can be considered to enhance therapeutic effects by combining different herbs and promoting synergistic interactions. Only by doing so can we better harness the potential of TCM and provide more options and solutions for the treatment of hepatic fibrosis. Finally, promoting the dissemination and promotion of TCM internationally to gain recognition and application worldwide. This will not only increase the visibility and influence of TCM but also attract more talents and resources to invest in TCM research and development.

All in all, the future development of TCM relies on efforts in scientific research, integration with modern medicine, international promotion, and talent cultivation. Only through strengthening research in TCM and continuously improving clinical practice can TCM better adapt to the needs of modern society, play an important role in the treatment of conditions like hepatic fibrosis, and make greater contributions to the cause of human health.
